# Polycyclic aromatic chains on metals and insulating layers by repetitive [3+2] cycloadditions

**DOI:** 10.1038/s41467-020-15210-2

**Published:** 2020-03-20

**Authors:** Alexander Riss, Marcus Richter, Alejandro Pérez Paz, Xiao-Ye Wang, Rajesh Raju, Yuanqin He, Jacob Ducke, Eduardo Corral, Michael Wuttke, Knud Seufert, Manuela Garnica, Angel Rubio, Johannes V. Barth, Akimitsu Narita, Klaus Müllen, Reinhard Berger, Xinliang Feng, Carlos-Andres Palma, Willi Auwärter

**Affiliations:** 1 Physics Department E20, Technical University of Munich, James-Franck-Str. 1, 85748 Garching, Germany; 2 Department for Molecular Functional Materials, Center for Advancing Electronics Dresden (cfaed), Faculty of Chemistry and Food Chemistry, Dresden University of Technology, Mommsenstr. 4, 01062 Dresden, Germany; 3 School of Physical Sciences and Nanotechnology, Yachay Tech University, 100119 Urcuquí, Ecuador; 4 Chemistry Department, College of Science, United Arab Emirates University (UAEU), P.O. Box 15551, Al Ain, United Arab Emirates; 5 Nano-Bio Spectroscopy Group and ETSF, Universidad del País Vasco, 20018 San Sebastián, Spain; 6 Max Planck Institute for Polymer Research, Ackermannweg 10, 55128 Mainz, Germany; 7 State Key Laboratory of Elemento-Organic Chemistry, College of Chemistry, Nankai University, 300071 Tianjin, China; 8 Instituto Madrileño de Estudios Avanzados en Nanociencia (IMDEA-Nanociencia), 28049 Madrid, Spain; 9 Max Planck Institute for the Structure and Dynamics of Matter, Luruper Chaussee 149, 22761 Hamburg, Germany; 10 Center for Free−Electron Laser Science and Department of Physics, University of Hamburg, Luruper Chaussee 149, 22761 Hamburg, Germany; 11 Organic and Carbon Nanomaterials Unit, Okinawa Institute of Science and Technology Graduate University, 1919-1 Tancha, Onna-son, Kunigami, Okinawa 904-0495 Japan; 12 Institute of Physical Chemistry, Johannes Gutenberg-University Mainz, Duesbergweg 10-14, D-55128 Mainz, Germany; 13 Institute of Physics, Chinese Academy of Sciences, 100190 Beijing, China

**Keywords:** Materials chemistry

## Abstract

The vast potential of organic materials for electronic, optoelectronic and spintronic devices entails substantial interest in the fabrication of π-conjugated systems with tailored functionality directly at insulating interfaces. On-surface fabrication of such materials on non-metal surfaces remains to be demonstrated with high yield and selectivity. Here we present the synthesis of polyaromatic chains on metallic substrates, insulating layers, and in the solid state. Scanning probe microscopy shows the formation of azaullazine repeating units on Au(111), Ag(111), and h-BN/Cu(111), stemming from intermolecular homo-coupling via cycloaddition reactions of CN-substituted polycyclic aromatic azomethine ylide (PAMY) intermediates followed by subsequent dehydrogenation. Matrix-assisted laser desorption/ionization (MALDI) mass spectrometry demonstrates that the reaction also takes place in the solid state in the absence of any catalyst. Such intermolecular cycloaddition reactions are promising methods for direct synthesis of regioregular polyaromatic polymers on arbitrary insulating surfaces.

## Abstract (web summary)

A critical milestone for the advancement of nanoscale organic circuitry is the fabrication of well-defined conjugated polymers on non-metal substrates. Here, the authors demonstrate extended polycyclic aromatic chains from repetitive cycloadditions which form not only on metals, but also on boron nitride layers and in the solid state.

## Introduction

Extended covalently bonded polyaromatic (polycyclic aromatic) systems hold great promise for molecular devices due to their increased structural and thermal stability and favorable electronic properties^1–8^. In many areas of technological applications and fundamental research, it is crucial to obtain covalently bonded fused ring systems (such as graphene and graphene nanoribbons) on insulating surfaces, to leverage device fabrication and integration of active layers onto dielectric substrates. In some cases, ex situ synthesis of such materials and subsequent processing and transfer onto insulating surfaces is possible. In situ synthesis, on the other hand, provides an alternative pathway towards functional nano-architectures and is expected to decrease costs, favor miniaturization, improve the purity and enhance the performance of polyaromatic frameworks. However, only few reaction types for aryl-aryl coupling have been realized on insulating surfaces^9–19^. The control of covalent coupling reactions on nonmetallic surfaces proves difficult due to the lack of metal-catalyzed carbon-hydrogen or carbon-halogen bond activation^20,21^. Coupling and, in particular, (aromatic) ring formation reactions on insulators thus often require harsher reaction conditions, the addition of catalysts, or external stimuli^16,18,22^. Such environments commonly lead to impurities and (randomly coupled) side products that remain on the surface, substantially hampering the quality of the fabricated architectures. Creation of atomically precise surface-supported organic materials for devices prompts catalyst-free polymerization^23^ and ring-formation reactions with high selectivity and preferably surface-independent reactivity. For instance, we recently introduced PAMY as a powerful halogen-free building block for the surface-assisted dimerization into a conjugated polycyclic hydrocarbon, namely pyrazine-embedded hexa-*peri*-hexabenzocoronene (N_2_-HBC)^24^. Therefore, further development of PAMY chemistry might grant access to previously unknown materials and in situ fabrication on device interfaces, such as solar cells and light-emitting devices^25^.

**Fig. 1:**
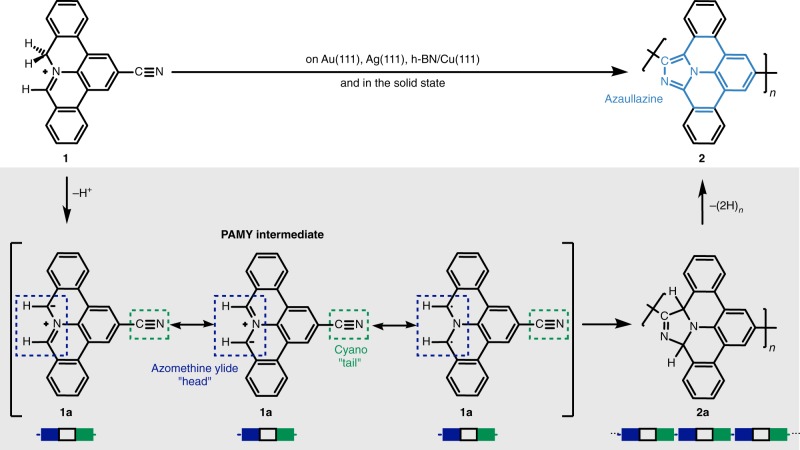
Conceptual route for the reported synthesis of polyaromatic azaullazine chains. The thermally induced head-to-tail coupling of **1** was observed on Au(111), Ag(111), h-BN/Cu(111), as well as in the solid state. Bottom: Reactive PAMY species **1a** as potential intermediates with affinity towards the cyano moiety, yielding intermolecular head-to-tail cycloaddition product **2a**. Subsequent dehydrogenation affords polyaromatic azaullazine chains **2**.

Here, we present a covalent intermolecular cycloaddition reaction of CN-functionalized PAMY with itself, into one-dimensional polyaromatic chains with azaullazine^26^ repeating units on metals, insulating layers, and in the solid state. Scanning tunneling microscopy (STM) and bond-resolved atomic force microscopy (AFM) with CO-functionalized tips demonstrates that on all investigated substrates, i.e., Au(111), Ag(111), and hexagonal boron nitride (h-BN) on Cu(111), phenanthridinium precursors predominantly form covalently coupled chains with repeating units of 1-azadibenzo[*d,k*]ullazine via the cycloaddition reaction^27,28^ between the cyano group and the azomethine ylide moiety, as well as subsequent dehydrogenation (see Fig. 1)^24,29–34^. The reaction did not proceed under solution reaction conditions, but was found to occur in the solid state as evidenced by MALDI measurements. This result suggests that the catalytic effect of the substrate is not crucial, and instead, the intrinsic reactivity of the molecules plays the dominant role, thus facilitating the cycloaddition reaction in the absence of catalysts, i.e., on insulating and inert substrates.

## Results

### Cycloaddition on metal substrates

**Fig. 2:**
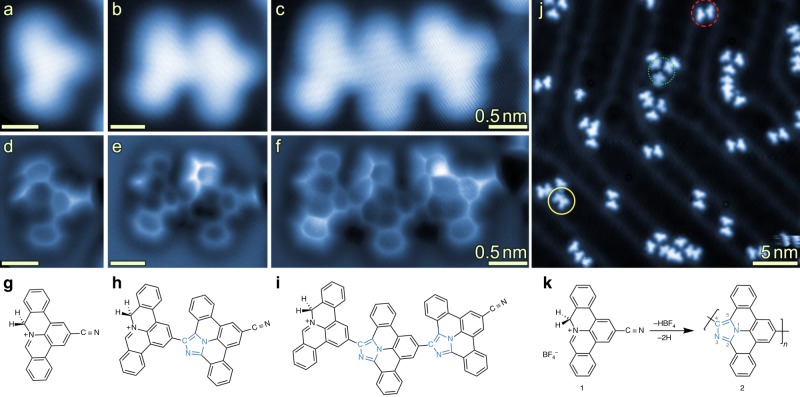
Head-to-tail coupling of **1** on a Au(111) substrate held at 600 K. **a**–**c** STM images of monomeric, dimeric, and trimeric species. **d**–**f** Bond-resolved AFM images of the monomer, dimer, and trimer shown in **a**–**c**. The images show the formation of an imidazole ring via cycloaddition of the –CN group of one monomer towards the phenanthridinium-derived azomethine ylide moiety of another monomer. **g**–**i** Chemical structures of the monomer, dimer, and trimer. The formed imidazole ring is highlighted in blue color. **j** Overview STM image shows a coexistence of covalently coupled (yellow solid circle) and noncovalently assembled (green dotted and red dashed circles) motifs. **k** Reaction scheme showing head-to-tail polymerization of **1** upon imidazole ring formation. Scan parameters: (**a**, **b**) Sample bias *V*_S_ = 20 mV, tunneling current setpoint *I* = 5 pA; (**c**) *V*_S_ = 20 mV, I = 20 pA; (**d**–**f**) *V*_S_ = 0 V, oscillation amplitude *A*_OSC_ = 40 pm, constant height; (**j**) *V*_S_ = 20 mV, *I* = 5 pA. Frequency shift ranges, dark to bright: (**d**) −10.2 to 6.7 Hz; (**e**) −10.1 to 3.1 Hz; (**f**) −9.9 to 7.2 Hz.

**Fig. 3:**
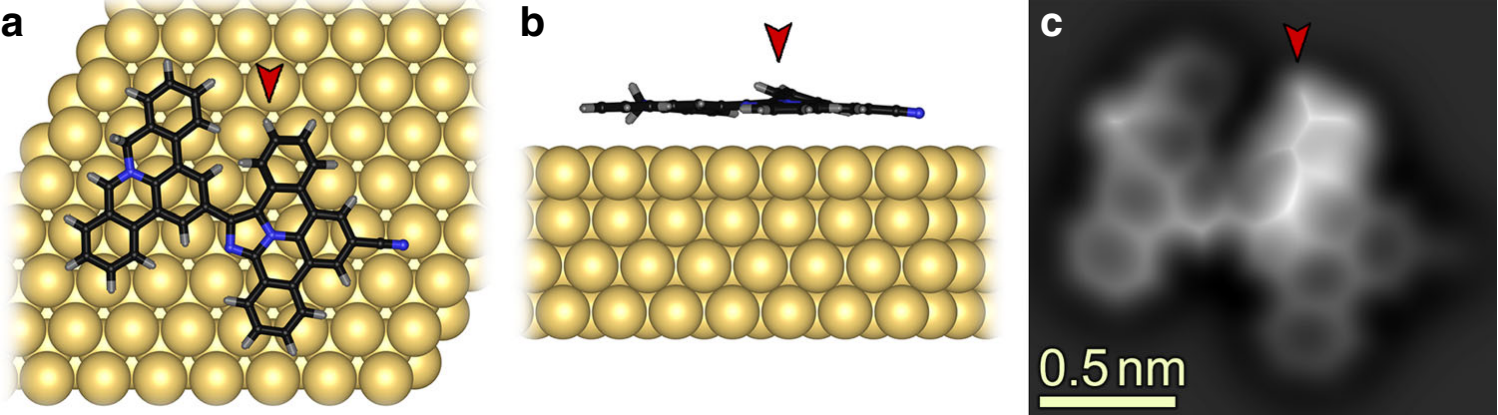
Theoretical modeling of a dimer on Au(111). (**a**) Top and (**b**) side views of the DFT-relaxed geometry of a dimer (as shown in Fig. 2h). One of the carbon atoms adjacent to the N-atom of the phenanthridinium moiety has two hydrogen atoms, pointing upwards and downwards, respectively. Furthermore, one of the phenyl rings of the right monomer (marked with red arrows in **a**–**c**) is bending upwards due to steric repulsions between the monomer units. **c** The simulated AFM image of the dimer shows a bright (more repulsive) contrast for the protruding hydrogen atom and the upwards-bending phenyl ring, in agreement with the contrast observed in the experimental image (Fig. 2e).

To afford repetitive intermolecular coupling we pursued a head-to-tail approach employing phenanthridinium precursors with a sterically accessible cyano-group (“tail”). The phenanthridinium precursor can form a reactive azomethine ylide group (“head”) in situ by proton cleavage^29,30,34^. The bifunctionalized precursor, 2-cyano-8*H*-isoquinolino[4,3,2-*de*]phenanthridin-9-ium tetrafluoroborate (**1**), was synthesized as described in our previous work^24^ and deposited onto a Au(111) surface held at 600 K. STM measurements revealed units that resemble the shape of the polyaromatic ring system of the cation and the cycloaddition products of head-to-tail coupled molecules (Fig. 2a–c). No evidence of the tetrafluoroborate counter-anions near their molecular units was found, suggesting that the anions or anion-derived species exhibit rather low sticking probability or/and are highly mobile on the surface (the anions could also not be detected when **1** was deposited onto a sample held at room temperature; see Supplementary Fig. 1 for more details)^35^. The STM images in Fig. 2b, c show increased local density of states (LDOS) between molecular units in the chains, in agreement with the formation of covalent bonds between the monomers. The resulting products were predominantly dimers and trimers. The proposed mechanism of the cycloaddition is shown in Fig. 1. Upon proton loss, the iminium group of precursor **1** forms the highly reactive azomethine ylide intermediate (**1a**) that exhibits zwitterionic and diradical reactivity^29,30,34^. The azomethine ylide intermediate **1a** can then undergo a reaction with the –CN group of another molecule, yielding a covalently coupled species **2a** with a five-membered ring. Subsequent dehydrogenation forms an imidazole ring leading to the product **2** with azaullazine units. Bond-resolved AFM imaging using CO-functionalized tips confirmed the formation of an imidazole ring as the linkage (Fig. 2e, f, h, i) between the monomers. In the AFM data, the area around the N1-atom of the imidazole ring appears darker, similar to previously reported nitrogen-doped nanoribbons on Au(111)^36,37^. The simulated AFM image of a dimeric structure (Fig. 3) agrees well with the measurements (Fig. 2e). The steric repulsion between the hydrogens at the chain’s ‘cove’ positions leads to a geometric distortion (Fig. 3a, b; Supplementary Fig. 2). The calculations indicate significant charge transfer of 0.76 e^−^ from the adsorbate to the substrate, resulting in a positively charged dimer (see Supplementary Fig. 3 for more details). At the employed reaction conditions, roughly half of the precursor molecules **1** underwent such covalent coupling reactions. Unreacted cyano-groups were identified to participate in noncovalent assemblies^38–40^ (dotted green and dashed red circles in Fig. 2j; see also Supplementary Figs. 4, 5).

**Fig. 4:**
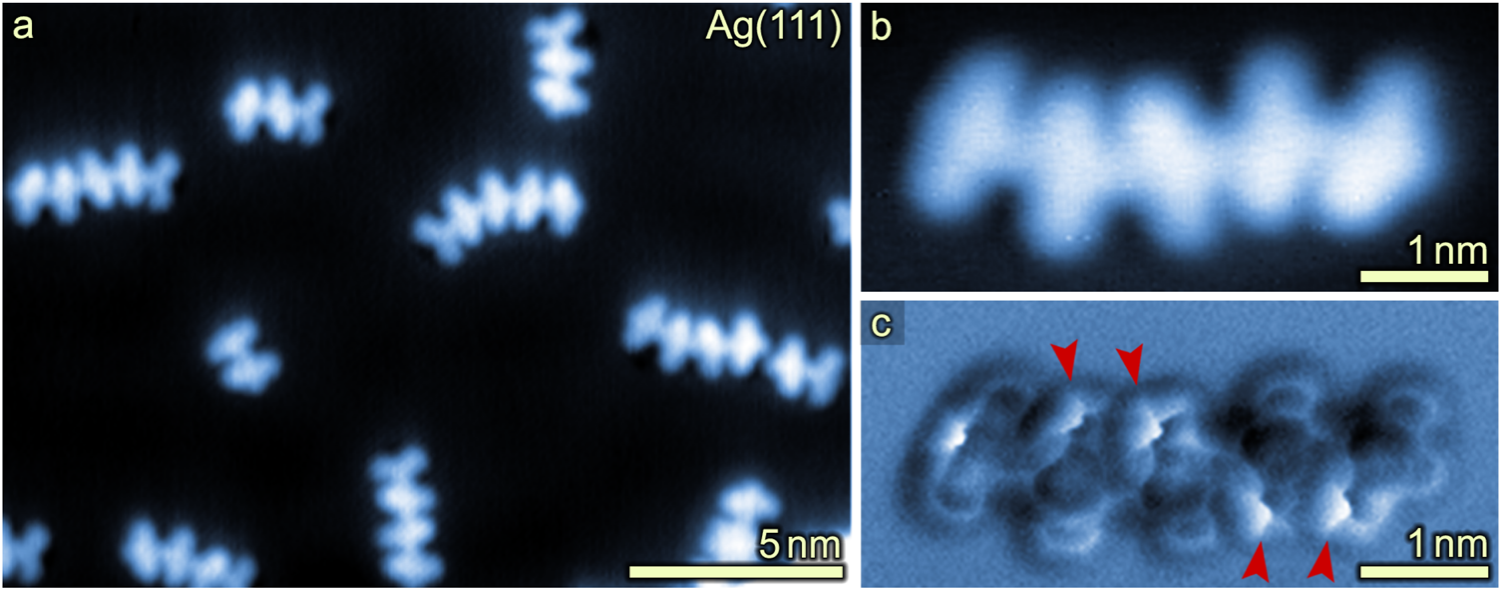
Head-to-tail coupling of 1 on Ag(111) held at 520 K. **a** Overview STM image after deposition of **1** onto a Ag(111) substrate held at 520 K shows polymerization towards one-dimensional molecular chains. (**b**) STM and (**c**) bond-resolved AFM image of a pentamer recorded with a CO-functionalized tip shows formation of imidazole rings between the monomer units—analogous to the coupling observed on Au(111). Red arrows highlight brightly imaged phenyl rings bending upwards due to steric repulsion. The terminal cyano group of the pentamer shown in **b** and **c** is cleaved off. Such loss of –CN has repeatedly been observed on Ag(111). Scan parameters: (**a**), (**b**) *V*_S_ = 100 mV, *I* = 10 pA, (**c**) *V*_S_ = 0 V, *A*_OSC_ = 80 pm, constant height. Frequency shift range: (**c**) −4.5 to 1.0 Hz.

Comparative studies on Ag(111) revealed that on this substrate moderate temperatures of 520 K analogously induce head-to-tail coupling via imidazole ring formation (Fig. 4). In contrast to Au(111), longer oligomers units were observed on Ag(111). Many of the terminal –CN groups were found to be abstracted (Fig. 4b, c) and thus non-covalently bonded assemblies via interaction of –CN groups were rarely observed. Owing to the single-bond connectivity between the chains and in-plane confinement, two possible orientations of a given constituent monomer in the chains are possible, giving rise to apparent (local) curvatures in the oligomers (Fig. 4b, c). Similar to Au(111), high-resolution AFM data of the coupled products on Ag(111) indicate that the phenyl rings in adjacent monomers are tilted upwards, due to steric hindrance with neighboring monomers (red arrows in Fig. 4c; phenyl rings bending upwards are imaged brighter)^41–45^.

### Cycloaddition on hexagonal boron nitride

**Fig. 5:**
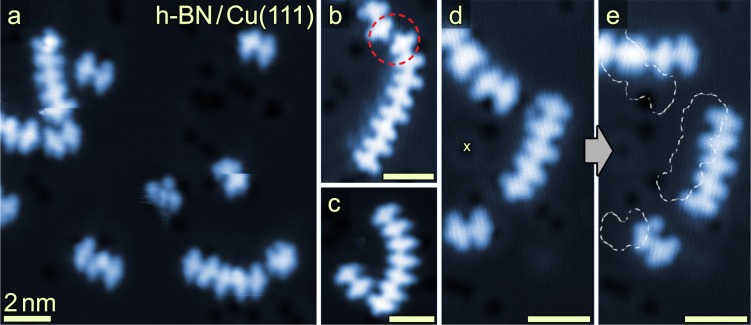
Oligomerization on h-BN/Cu(111) after deposition of **1** onto the surface held at 500 K. **a** STM overview image shows coupled molecular units, analogous to the cycloaddition products observed on Au(111) and Ag(111). Unidentified species are also observed, cf. image’s center and Table 1. **b**, **c** Close-up STM images reveal the formation of chains of up to eight monomer units. Non-covalent aggregation with antiparallel alignment of the –CN dipoles of a dimer and an octamer is marked by a red dashed circle in **b**. **d** STM image of oligomer chains. **e** After application of a voltage pulse (*V*_S_ = −2.5 V, *I* = 100 pA, *t* = 1 s) at the position marked with a yellow cross in **d** a shift of the positions of the chains is observed. The chains remain intact and each chain is shifted as a whole. The positions of the chains before the voltage pulse are marked with white dashed traces (data in **a**, **c**–**e** recorded after post-annealing the sample to 580 K). Scan parameters: (**a**) *V*_S_ = 1.10 V, I = 50 pA, (**b**) *V*_S_ = 0.66 V, *I* = 60 pA, (**c**) V_S_ = 1.0 V, I = 50 pA; (**d**), (**e**) *V*_S_ = 1.7 V, *I* = 24 pA.

The chemical reactivity of the phenanthridinium precursors **1** on h-BN layers is strikingly similar to what was observed on the noble metals. h-BN is a chemically inert insulator that can be grown as a monolayer on Cu(111)^45–47^. Deposition of **1** onto a h-BN/Cu(111) substrate held at 500 K similarly resulted in head-to-tail coupling of the precursor molecules and formation of chains as shown in the STM images in Fig. 5 (the samples were annealed up to 580 K after the deposition of **1**). Dimeric structures, as well as longer chains of up to eight covalently coupled units were present on the surface. No unreacted monomers were found, likely due to facile molecular desorption of molecular species from h-BN at elevated temperatures. STM data of the observed chains on h-BN/Cu(111) resemble the images of the oligomers on Au(111) and Ag(111) (compare Figs. 2, 4, and 5). As observed on the metal surfaces, the increased LDOS between molecular subunits on h-BN/Cu(111) indicates the formation of chemical bonds between the monomer units. The distances between the centers of neighboring repeating units in a chain were found to be 0.8 nm on all substrates. Bond-resolved AFM measurements were hampered by the facile movement of the molecules, as well as challenges in obtaining a CO-functionalized tip on h-BN/Cu(111). Instead, we have conducted lateral manipulation experiments to further strengthen the evidence for the chemical coupling of the molecular units. Figure 5d, e shows data of molecular chains before and after the application of a voltage pulse (*V*_S_ = −2.5 V, *I* = 100 pA, *t* = 1 s) in close vicinity of the chains (see also Supplementary Fig. 6). The voltage pulse induced a lateral displacement of the chains. Importantly, each of the chains moved as one unit, as expected for covalently bonded oligomeric chains.

A peculiarity of the reported head-to-tail coupling reaction concerns its selectivity on diverse substrates. Head-to-tail coupling, although abundantly observed, competes with the head-to-head dimerization of the phenanthridinum-derived azomethine ylide^24^ and with spurious motifs mostly consisting of the head-to-side products (see Supplementary Fig. 7). The side reactions can be explained by formation of the reactive azomethine ylide and its homo-coupling on metal surfaces, as well as less controlled diradical reaction pathways^24,29–34^. Table 1 provides an overview of the reaction selectivity on Au(111), Ag(111), and h-BN/Cu(111). The predominance of head-to-tail coupling on all the investigated surfaces suggests that the catalytic activity of the substrate is not essential to mediate this reaction. In particular, the selectivity of the head-to-tail coupling on h-BN/Cu(111) reached ~80%. The high selectivity on the investigated substrates indicates that the coupling is promoted by the intrinsic propensity of the phenanthridinium/azomethine moiety to engage in reactions with cyano groups.

**Table 1:** Coupling selectivity on Au(111), Ag(111), and h-BN/Cu(111) in the temperature range of 500 and 600 K based on statistical analysis of 400 reaction events.

Motif	Au(111)	Ag(111)	h-BN/Cu(111)
Head-to-tail	62%	97%	76%
Head-to-head	<1%	1%	4%
Head-to-side	38%	<1%	<1%
Unidentified	<1%	<1%	∼20%

### Cycloaddition in the solid state

**Fig. 6:**
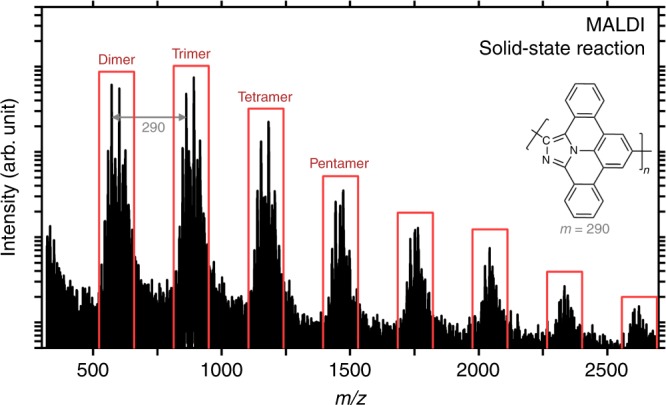
Solid state oligomerization of **1**. The MALDI-ToF mass spectrum after thermal annealing of **1** to 520 K for 72 h under vacuum (10^−2^ to 10^−1^ mbar) shows distinct peak groups indicative of intermolecular coupling upon formation of oligomers (dimers, trimers, tetramers, etc.). The separation of 290 m/z between these peak groups corresponds to the mass of one monomer unit (C_21_N_2_H_10_, chemical structure in the inset).

To provide unambiguous evidence for polymerization in the absence of catalysts, we demonstrate that thermally induced oligomerization of **1** occurs in the solid state. Precursor **1** was studied by MALDI Time-of-Flight (ToF) mass spectrometry before and after solid state heating in powder form under vacuum (glass ampoule; 10^−2^ to 10^−1^ mbar) to 520 K for 72 h. After this heat treatment, the main features of the recorded spectrum evidence oligomeric reaction products (Fig. 6). Notably, there are distinct groups of peaks separated by *m*/*z* ratios of 290 and up to octameric species. This exact m/z ratio corresponds to the mass of one dibenzoazaullazine unit (see the chemical structure in the inset in Fig. 6). The dominating signals appear to correspond to azaullazine oligomers with carboxylic group (–COOH) moieties (see also Supplementary Discussion), e.g., dimer–COOH (exp.: *m*/*z* = 602.1923, calc. for C_42_H_24_N_3_O_2_: *m*/*z* = 602.1868), trimer–COOH (exp.: *m*/*z* = 892.2703, calc. for C_63_H_34_N_5_O_2_: *m*/*z* = 892.2713), tetramer–COOH (exp.: *m*/*z* = 1182.3580; calc. for C_84_H_44_N_7_O_2_: *m*/*z* = 1182.3556), which can be explained by hydrolysis of cyano (–CN) terminations at the reaction conditions due to ambient preparation of the capsule. Note that the reaction did neither proceed under solution conditions which favor dipolar additions^29,30,34^ nor under dimethyl sulfoxide reflux.

### Reaction energies

**Fig. 7:**
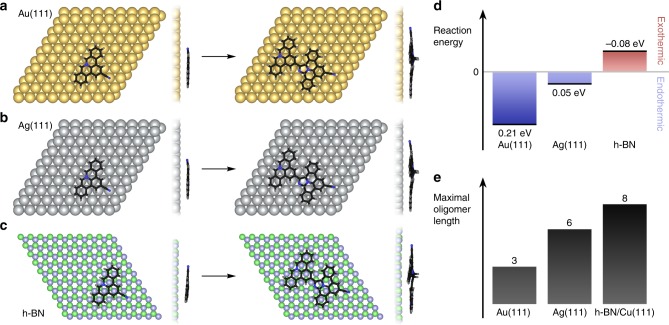
DFT reaction study of the dimerization step of **1a**. Top and side views of **1a** and **2a**-type dimers on (**a**) Au(111), (**b**) Ag(111), and (**c**) h-BN. **d** The computed reaction energies show that the reaction step is endothermic on Au(111) and Ag(111) and slightly exothermic on h-BN. **e** Maximum oligomer lengths observed experimentally on Au(111), Ag(111), and h-BN/Cu(111).

To explore the influence of the different substrates on the reaction yield and oligomer length, we investigated via DFT the reaction energies for the coupling of two species of **1a** towards the dimer **2a** (Fig. 1, gray) on Au(111), Ag(111) and h-BN (Fig. 6a–d). We find that this dimerization step is endothermic on the metal substrates (ΔE = 0.21 eV on Au(111), ΔE = 0.05 eV on Ag(111)) and exothermic (ΔE = −0.08 eV) on h-BN. The strong difference in the reaction energies—which according to the Bell-Evans-Polanyi (BEP) principle correlate with reaction barriers^48,49^—between Au(111) and h-BN is reflected in the head-to-tail coupling yield and oligomer length: the reaction yield and oligomer length are substantially higher on h-BN/Cu(111) (see Fig. 6e and Table 1) despite the lower employed temperature of 500 K (vs. 600 K for Au(111)). Analysis of the DFT energy components reveals that the origin of the energy discrepancy is twofold. First, the formal biradical intermediates **1a** are stabilized on Au(111) as evidenced by the high adsorption energy of 5.33 eV for two non-interacting **1a** molecules. Second, the dimer **2a** (Fig. 7a) features two sp^3^ carbons which are strongly strained on Au(111). In contrast, the weakly interacting h-BN/Cu(111) template neither promotes additional radical stabilization (the adsorption energy of two independent **1a** molecules amounts to 2.7 eV) nor considerable product strain. Our calculations furthermore predict that the reaction is exothermic in the gas phase (ΔE = −0.83 eV) due to the absence of geometry distortions associated with strong adsorption on a substrate. Since planarization and biradical stabilization are expected to be less eminent in the solid state, formation of longer oligomer chains becomes favorable (see Fig. 6).

## Discussion

The comparative studies described above serve as a benchmark for the development of new types of polymer and N-doped material fabrication strategies directly at interfaces. Surface-independent chemical coupling will generally depend on reaction types that can proceed in the solid state. The synthesis of planar polyaromatic frameworks (such as graphene nanoribbons—see also Supplementary Fig. 8) will often proceed via three-dimensional and/or radical-like intermediates such as **1a** and **2a**. The yield of such reactions can be hampered by metallic substrates due to strong interaction with the intermediates. On Au(111) for example, the reactants were seen to form metallosupramolecular assemblies via their unreacted cyano groups (see Supplementary Fig. 4). Furthermore, catalytically active metal substrates can facilitate alternative reaction pathways, such as the undesired head-to-side coupling on Au(111) (see Table 1) and cleavage of the terminal –CN groups during thermal annealing on Ag(111) (cf. Fig. 4). Interestingly, the longest visualized oligomers were octameric species on h-BN/Cu(111). Under ultra-high vacuum conditions (UHV), desorption of the reactants is expected to limit the maximum chain length on h-BN/Cu(111). Thus, an important future goal will be to improve the coupling vs. desorption probability of the precursor molecules on h-BN/Cu(111) and other inert substrates, to realize longer polymers and higher surface coverage for applications. Apart from larger precursors, this can also be accomplished by employing higher molecular density/flux at high temperatures to maintain proper monomer mobility^50^. One challenge of this approach is the introduction of potential contaminants such as water, which—as shown above—can be detrimental to the polymerization reaction. Prolonged drying and the use of carrier gases can mitigate this effect. We have tested the feasibility of this strategy by a second solid state polymerization reaction in a vacuum furnace setup under argon carrier gas, which yielded longer chains beyond 20 coupled units as observed in MALDI data (see Supplementary Fig. 16). Overall, our insights for the careful optimization of synthesis protocols, in combination with precursor engineering, are expected to enable extended and functional polymeric nanoarchitectures.

In summary, we have shown a paradigmatic on-surface oligomerization which can be employed to fabricate polyaromatic chains with azaullazine-units on Au(111), Ag(111), h-BN/Cu(111), and in the solid state. The design and functionality of the in situ generated PAMY precursors allow the direct catalyst-free synthesis of polyaromatic chains and ribbons^33,34,51,52^. The intermolecular head-to-tail coupling of azomethine ylide and –CN group is the dominant reaction pathway in all cases. We expect that monomer engineering will open up the possibility to fabricate long conjugated polymers and graphene nanoribbons, placing azomethine ylide and derived chemistry in a privileged position for the implementation of on-surface polymerization at insulating interfaces.

## Methods

### Scanning probe microscopy

The precursor 2-cyano-8*H*-isoquinolino[4,3,2-*de*]phenanthridin-9-ium tetrafluoroborate (**1)** was synthesized according to literature procedure^24^. The precursor **1** was sublimated from a Knudsen cell heated to ~750 K onto Au(111), Ag(111), and h-BN/Cu(111) in ultra-high vacuum (at background pressures around 10^−9^ mbar). Typical evaporation times were around 10 min. The metal surfaces were cleaned by cycles of argon ion sputtering and annealing. h-BN on Cu(111) was grown from borazine precursors^45,47^. Scanning probe measurements were performed using CreaTec STM/AFM instruments under ultra-high vacuum conditions (p ≈ 10^−10 ^mbar) at a temperature of 5 to 6 K. STM images were recorded in constant current mode. AFM imaging was performed in frequency modulation mode using a qPlus sensor (resonance frequency ≈30 kHz, typical Q values of 70,000, typical oscillation amplitudes of 40 pm). AFM scans were acquired at constant height (i.e., with the z-feedback switched off) at a sample bias of *V*_S_ = 0 V. For tip functionalization carbon monoxide (CO) was dosed onto the cold sample and transferred to the tip^53,54^. AFM images are unprocessed. STM images were subjected to standard corrections, i.e., plane-subtraction, as well as global brightness/contrast adjustments in selected cases to ensure comparable contrast across all shown images. The images were processed using the Nanonis image viewer software, as well as WSxM^55^.

### Mass spectrometry

The precursor **1** was pressed into a pill form (Specac compression device, seven tons) and put into glass ampoule. The ampoule was sealed under vacuum after three-pump-thaw cycles (~10^−2 ^mbar) and then heated to 520 K for 72 h. After cooling to room temperature, the crude mixture was analyzed by MALDI-ToF mass spectrometry measurements without further purification. MALDI-ToF MS spectra were recorded on a Bruker Autoflex Speed MALDI-ToF MS (Bruker Daltonics, Bremen, Germany) with a tetracyanoquinodimethane (TCNQ) as the matrix. For the thermal polymerization in a vacuum furnace, a powder sample of the precursor was placed inside a tube furnace. After evacuation of the tube, a gas flow consisting of 100 sccm hydrogen and 500 sccm argon was introduced, raising the pressure in the tube to 1.5 mbar. The furnace was kept for 15 min at room temperature for removing residual moisture and oxygen from the system, and then heated to 450 °C within 14 min and kept at this temperature for 31 min. The sample was cooled down to a room temperature under the gas flow.

### Density functional theory

All slab calculations used the PBE^56^ exchange-correlation functional. We included van der Waals (vdW) corrections the Grimme’s D3 method^57^. We investigated the low-coverage physisorption on a 9 × 9 × 4 Au(111) and Ag(111) slabs (with Cartesian coordinates derived from the experimental lattice constants 4.078 Å and 4.085 Å, respectively) and on a 10 × 10 h-BN monolayer (B-N bond length 1.4457 angstroms). All atoms were relaxed for the latter system, whereas only the adsorbate and top Au layer were relaxed for metal substrates. Geometry relaxations were stopped once all ionic forces fell below 0.025 eV/Å. The DFT reaction energies, i.e., the differences between the energies between the dimer (**2a**) and monomer (**1a**) species, were calculated on frozen metal surfaces and on a relaxed h-BN sheet. These calculations were performed with the QS module^58^ of the CP2K code^59^ and with the VASP code^60–62^. The charge transfers were computed via the Bader analysis code by Henkelman et al.^63–65^. STM images were simulated under the Tersoff-Hamann s-wave tip approximation^66^. AFM simulations were performed with the “Probe Particle Model” of Hapala et al.^67,68^. Full computational details are given in the Supplementary Methods.

## Supplementary information

Supplementary Information

## Data Availability

The data that support the findings of this study are available from the corresponding authors on reasonable request.
