# Author Correction: Polycyclic aromatic chains on metals and insulating layers by repetitive [3+2] cycloadditions

**DOI:** 10.1038/s41467-020-17857-3

**Published:** 2020-08-04

**Authors:** Alexander Riss, Marcus Richter, Alejandro Pérez Paz, Xiao-Ye Wang, Rajesh Raju, Yuanqin He, Jacob Ducke, Eduardo Corral, Michael Wuttke, Knud Seufert, Manuela Garnica, Angel Rubio, Johannes V. Barth, Akimitsu Narita, Klaus Müllen, Reinhard Berger, Xinliang Feng, Carlos-Andres Palma, Willi Auwärter

**Affiliations:** 1 Physics Department E20, Technical University of Munich, James-Franck-Str. 1, 85748 Garching, Germany; 2 Department for Molecular Functional Materials, Center for Advancing Electronics Dresden (cfaed), Faculty of Chemistry and Food Chemistry, Dresden University of Technology, Mommsenstr. 4, 01062 Dresden, Germany; 3 School of Physical Sciences and Nanotechnology, Yachay Tech University, 100119 Urcuquí, Ecuador; 4 Chemistry Department, College of Science, United Arab Emirates University (UAEU), P.O. Box 15551, Al Ain, United Arab Emirates; 5 Nano-Bio Spectroscopy Gr oup and ETSF, Universidad del País Vasco, 20018 San Sebastián, Spain; 6 Max Planck Institute for Polymer Research, Ackermannweg 10, 55128 Mainz, Germany; 7 State Key Laboratory of Elemento-Organic Chemistry, College of Chemistry, Nankai University, 300071 Tianjin, China; 8 Instituto Madrileño de Estudios Avanzados en Nanociencia (IMDEA-Nanociencia), 28049 Madrid, Spain; 9 Max Planck Institute for the Structure and Dynamics of Matter, Luruper Chaussee 149, 22761 Hamburg, Germany; 10 Center for Free−Electron Laser Science and Department of Physics, University of Hamburg, Luruper Chaussee 149, 22761 Hamburg, Germany; 11 Organic and Carbon Nanomaterials Unit, Okinawa Institute of Science and Technology Graduate University, 1919-1 Tancha, Onna-son, Kunigami, Okinawa 904-0495 Japan; 12 Institute of Physical Chemistry, Johannes Gutenberg-University Mainz, Duesbergweg 10-14, D-55128 Mainz, Germany; 13 Institute of Physics, Chinese Academy of Sciences, 100190 Beijing, China

**Keywords:** Materials chemistry

Correction to: *Nature Communications*10.1038/s41467-020-15210-2, published online 20 March 2020.

The original version of this Article omitted some funding sources in the Acknowledgements. The correct version of the Acknowledgments is:

“This work was financially supported by the European Research Council Consolidator Grant NanoSurfs (no. 615233), the Advanced Grant (no. 694097), the Horizon 2020 research and innovation program 2D ink (no. 664878), and the National Science Foundation of China (no. 11974403 and Sino-German Project no. 51761135130). W.A. acknowledges funding by the DFG via a Heisenberg professorship. M.R., R.B., and X.F. thank the German Research Foundation (DFG) within the Cluster of Excellence “Center for Advancing Electronics Dresden (cfaed)” and EnhanceNano (No. 391979941). A.P.P. and A. Rubio thank the Cluster of Excellence “Advanced Imaging of Matter (AIM)” and Grupos Consolidados (IT1249-19). M.G. acknowledges funding by the H2020-MSCA-IF−2014 program under GA no. 658070 (2DNano).”

Which replaces the previous incorrect version:

“This work was financially supported by the European Research Council Consolidator Grant NanoSurfs (no. 615233), the Horizon 2020 research and innovation program 2D ink (no. 664878) and the National Science Foundation of China (no. 11974403 and Sino-German Project no. 51761135130). W.A. acknowledges funding by the DFG via a Heisenberg professorship. M.R., R.B., and X.F. thank the German Research Foundation (DFG) within the Cluster of Excellence “Center for Advancing Electronics Dresden (cfaed)” and EnhanceNano (No. 391979941). M.G. acknowledges funding by the H2020-MSCA-IF−2014 program under GA no. 658070 (2DNano).”

This has now been corrected in both the PDF and HTML versions of the Article.

