# Supplementary material for: Polycyclic aromatic chains on metals and insulating layers by repetitive [3+2] cycloadditions
Source: Nat Commun. 2020 Mar 20;11:1490. doi: 10.1038/s41467-020-15210-2 (PMC7083871; doi:10.1038/s41467-020-15210-2)
Supplement: Supplementary file 1 — Supplementary Information [file 41467_2020_15210_MOESM1_ESM.pdf]

Supplementary Information

Polycyclic aromatic chains on metals and insulating layers by repetitive [3+2]cycloadditions

Riss et al.

Contents

Supplementary Figures 3
Supplementary Discussion 11
Supplementary Methods 19
Supplementary References 20

Supplementary Figures

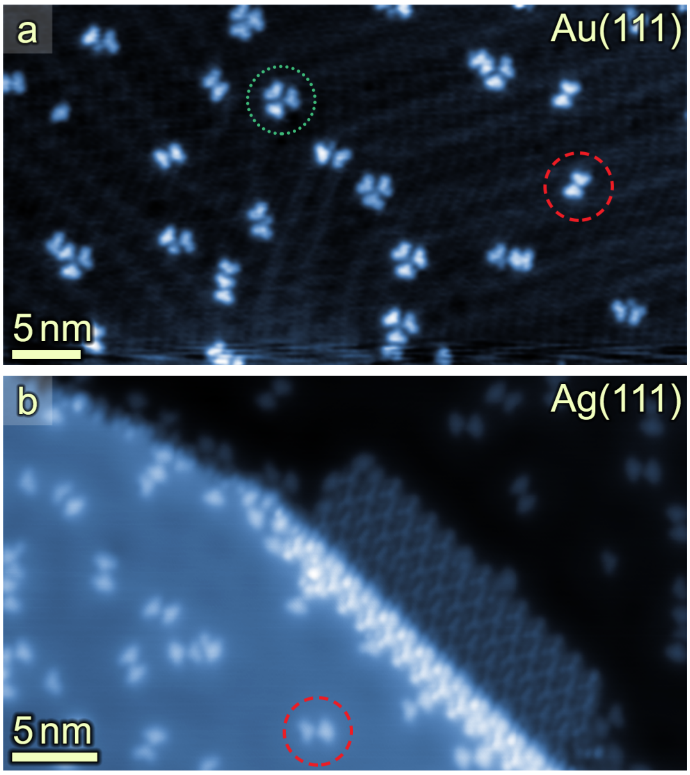

Supplementary Figure 1. Deposition of **1** before annealing. STM images of monomer deposition onto **(a)** Au(111) and **(b)** Ag(111) held close to room temperature show unreacted monomeric species, most of which participate in noncolavent interactions (dotted green and dashed red circles; see also Supplementary Figures 4 and 5). The STM image in (b) additionally shows the formation of an island of monomers at the step edge. While oligomerization can occur in the crucible during the evaporation procedure (similar to what we observed for the solid state reaction), these experiments show that the coupled species are not deposited onto the surfaces at the applied evaporation parameters.

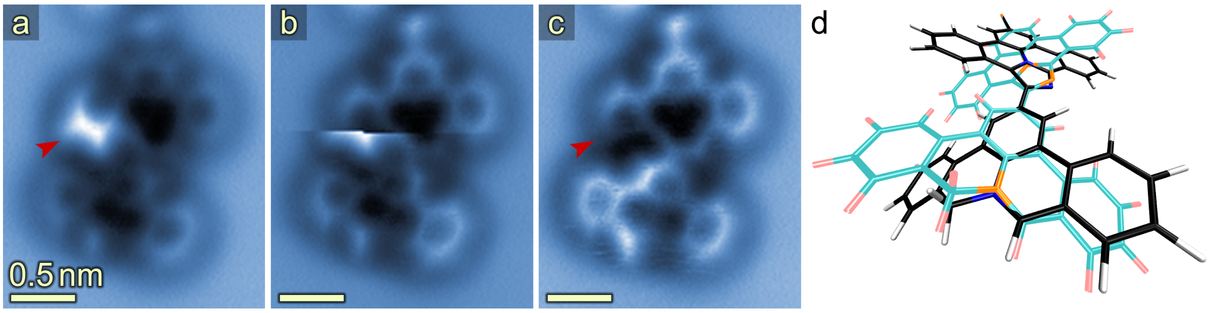

Supplementary Figure 2. Tip-induced switching between two conformers of a dimer on Au(111). **(a)** Constant-height AFM image of a covalently coupled dimer. The bright (more repulsive) contrast in the image is caused by upwards-bending of the phenylene ring marked with a red arrow in (a). **(b)** During the AFM scan a change of the contrast associated with this phenylene ring is observed. **(c)** The adsorption geometry of the dimer has changed. A darker (less repulsive) contrast (red arrow) indicates that the phenylene ring is now bending downwards, *i.e.* towards the surface. **(d)** Structural models of two conformers with dihedral angles between the monomers of +30.71° and -31.46° based on gas phase DFT calculations. The two gas-phase conformers are isoenergetic with an energy barrier of 0.32 eV between them (calculated at the B3LYP-D3/def2-TZVP level). Similarly, experiments show significant quantities of both conformers directly after deposition onto Au(111). Scan parameters: V_S = 0 V, A_{OSC} = 80 pm, constant height. Slow scan direction: bottom to top. Frequency shift ranges: (a) -13.5 to -7.1 Hz, (b) -13.1 to -6.8 Hz, (c) -13.3 to 7.8 Hz.

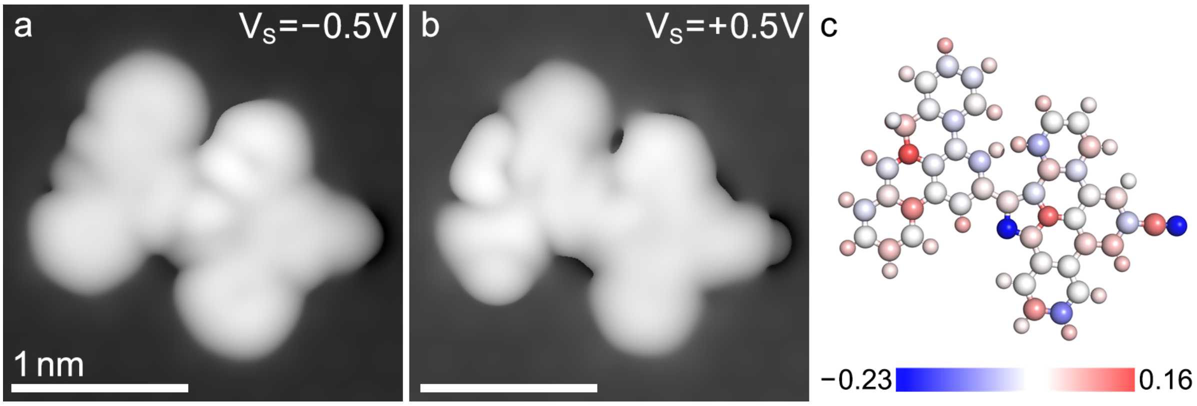

Supplementary Figure 3. Calculated electronic structure of a positively charged dimer on Au(111). Simulated STM images of a dimer adsorbed on Au(111) for sample biases of **(a)** V_S = -0.5 V and **(b)** V_S = 0.5 V. In agreement with experiment, the simulations show increased LDOS between the molecular subunits due to the formation of covalent bonds. **(c)** Bader charge analysis of the positively charged system shows that all the positive charge of 1 e remains localized in the molecular adsorbate. The nitrogen atoms of the unreacted and the reacted phenanthridinium moieties carry positive charges of 0.16 and 0.14 e, respectively. The surface atoms have been omitted for clarity. Furthermore, calculations of an electronically neutral system (not shown) indicate charge transfer of 0.76 e^- from the molecule to the substrate, similarly resulting in a positively charged adsorbate.

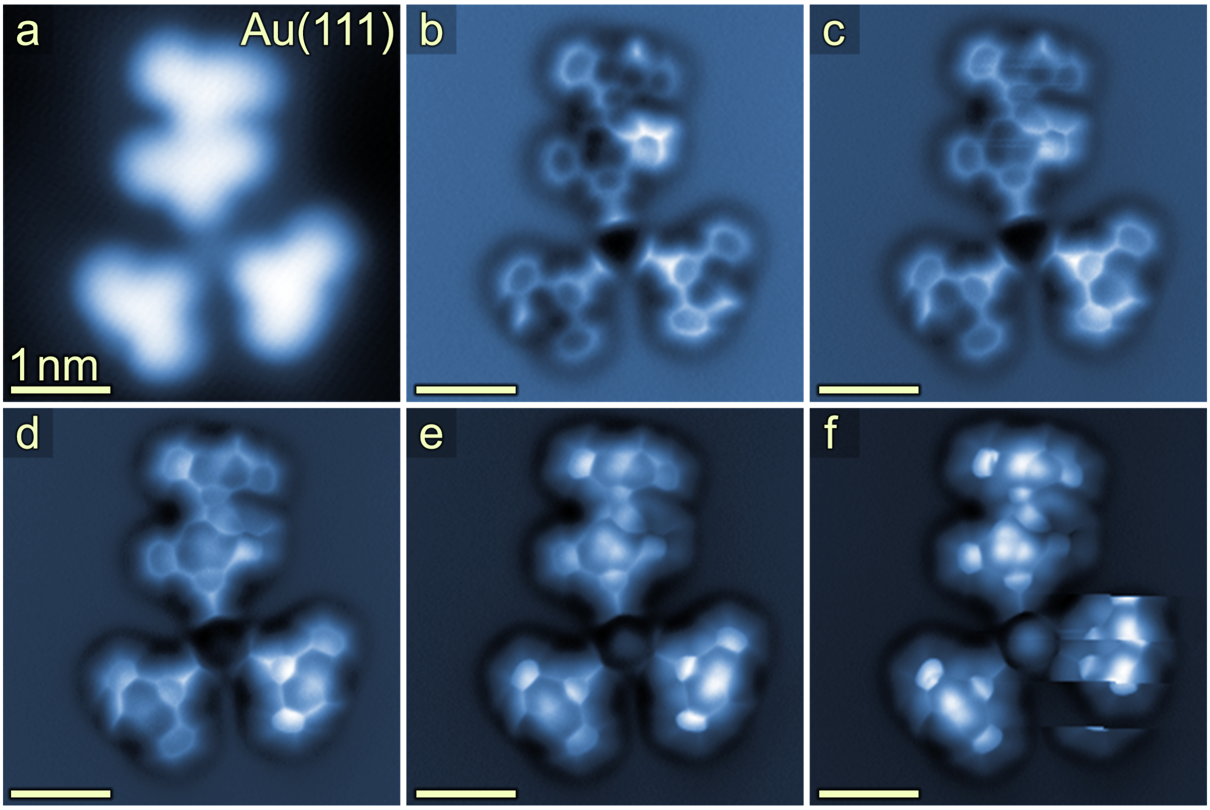

Supplementary Figure 4. Metal-organic coordination on Au(111) via interaction of terminal cyano groups with adatoms. **(a)** STM image shows terminal –CN groups of two monomers and one dimer pointing towards a common center – presumably a Au adatom.[1–10] **(b)-(f)** Series of bond-resolved AFM images (with a CO-functionalized tip) at different heights. The tip-sample distance was decreased by 20 pm after each scan. A bright protrusion, which is likely associated with a Au adatom or relaxed surface atom, becomes apparent at smaller tip-sample distances. Scan parameters: (a) V_S = 20 mV, I = 5pA; (b)-(f) V_S = 0 V, A_{OSC} = 40 pm, constant height. Frequency shift ranges: (b) -10.0 to 3.1 Hz; (c) -10.2 to 6.7 Hz; (d) -9.8 to 11.2 Hz; (e) -9.3 to 16.7 Hz; (f) -9.8 to 21.7 Hz.

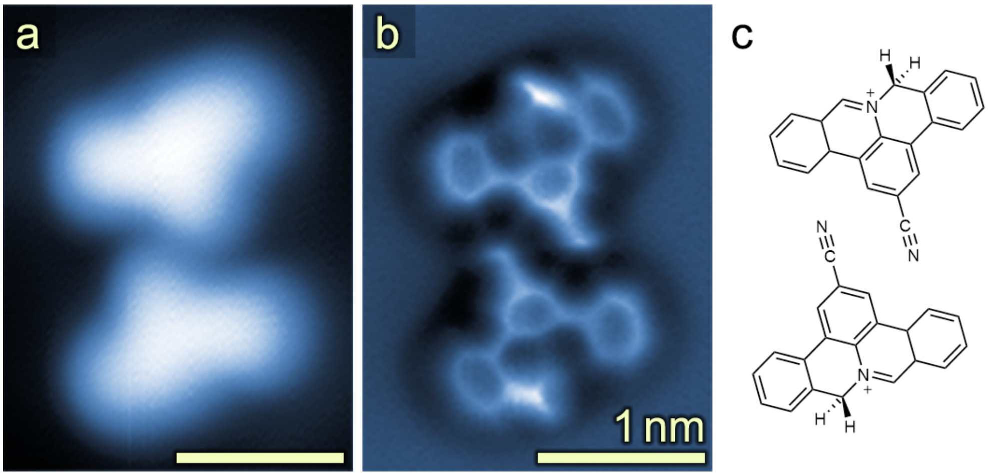

Supplementary Figure 5. Noncovalent interaction of monomers via their cyano groups on Au(111). **(a)** STM image of two monomers in an antiparallel alignment. **(b)** Bond-resolved AFM image showing noncovalently interacting molecules with antiparallel alignment of their cyano groups. **(c)** Chemical structure model of the two monomers. The structure suggests stabilization by hydrogen bonding, as well as dipole-dipole interactions.[1–4,7–9,11–19] Scan parameters: (a) V_S = 20 mV, I = 5 pA; (b) V_S = 0 V, A_{OSC} = 40 pm, constant height. Frequency shift range: (b) -10.2 to 1.5 Hz.

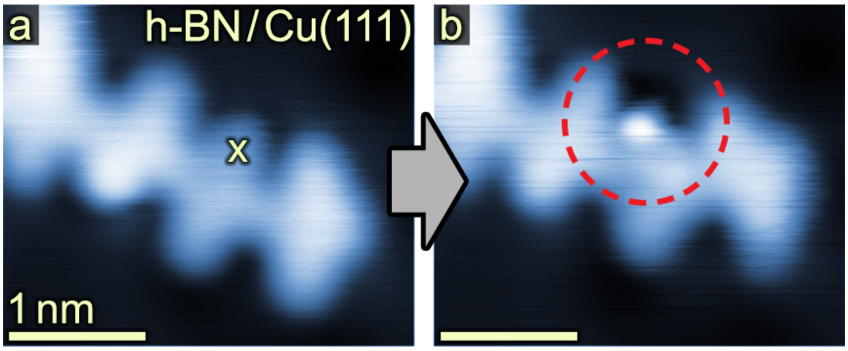

Supplementary Figure 6. Tip-induced switching between two conformers of a oligomer chain on h-BN/Cu(111). **(a)** Close-up STM image of a chemically coupled oligomeric chain. **(b)** Application of a voltage pulse (V_S = 3.6V, I = 50pA, few seconds) at the position marked with a yellow cross in (a) changes the local STM contrast (marked with a red dashed circle), which indicates an altered adsorption geometry – likely due to out-of-plane bending of the respective phenylene ring. Such geometry changes have also been observed on the Au(111) surface (see Supplementary Figure 2 for more details). The images were taken after post-annealing to 630 K. Scan parameters: V_S = 1.4V, I =50 pA.

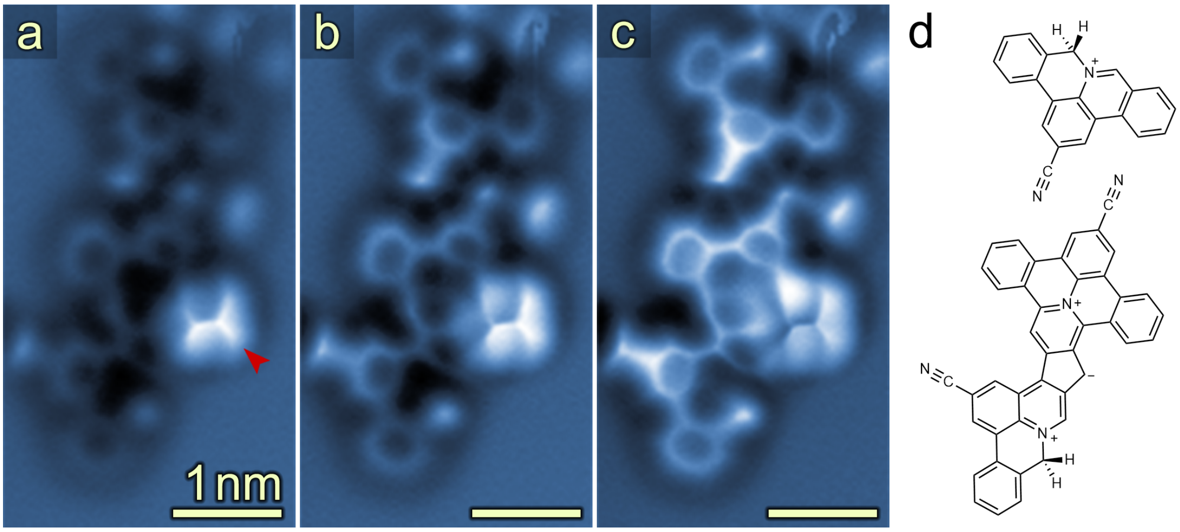

Supplementary Figure 7. Head-to-side coupling on Au(111). **(a)-(c)** Series of bond-resolved AFM scans with a CO-functionalized tip at different tip-sample distances shows head-to-side coupling of monomers. The tip height was decreased by 20 pm after each scan. The bright contrast in the AFM images (marked with a red arrow in (a)) is due to a geometric distortion of the molecule, *i.e.* upwards-bending of the phenylene ring. **(d)** The tentative chemical structure model of the product as derived from the AFM scans suggests a ring rearrangement.[20–22] Scan parameters: V_S = 0 V, A_{OSC} = 40 pm, constant height. Frequency shift ranges: (a) -9.8 to 4.2 Hz, (b) -9.1 to 2.7 Hz, (c) -9.0 to 3.1 Hz.

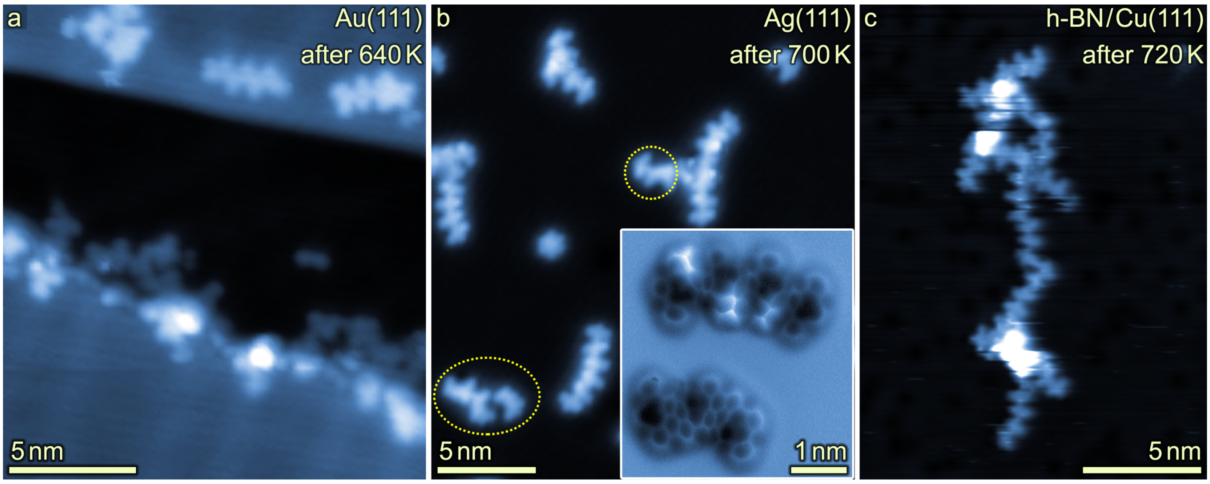

Supplementary Figure 8. Annealing of oligomer chains on Au(111), Ag(111) and h-BN/Cu(111). **(a)** STM image after deposition of **1** onto Au(111) and heating to 640 K shows coupled agglomerates via head-to-side and other (unidentified) motifs. **(b)** Deposition of **1** onto Ag(111) and annealing to 700 K induced cyclodehydrogenation of the chains upon formation of flattened nanoribbon-like structures (dashed yellow ellipses). The AFM image in the inset shows a chain of four coupled units on the top and a nanoribbon-like structure on the bottom. The nanoribbon was formed by intramolecular cyclodehydrogenation of a chain that consisted of three coupled units. In contrast to the oligomer chain, the nanoribbon is a planar structure as indicated by its rather uniform contrast in AFM. **(c)** STM image after deposition of **1** onto h-BN/Cu(111) and annealing to 720 K shows no evidence for cyclodehydrogenated and flattened products. However, an increased number of side reactions was observed leading to the formation of branched structures. Scan parameters: (a) V_S = 0.02 V, I = 5 pA; (b) V_S = 0.2 V, I = 20 pA; inset in (b) V_S = 0 V, A_{OSC} = 80 pm, constant height. (c) V_S = 1.2 V, I = 50 pA. Frequency shift range: inset in (b) -5.4 to 0.5 Hz.

Supplementary Discussion

The HR-MALDI-ToF measurement exhibits distinct peak groups in a distance of m/z = 290. The distance of m/z = 290 can be assigned to a dibenzoazaullazine motif. The main peaks for the dimers, trimers and tetramers are shown in Supplementary Figures 9-14, along with the corresponding tentative structures and the simulated mass spectra.

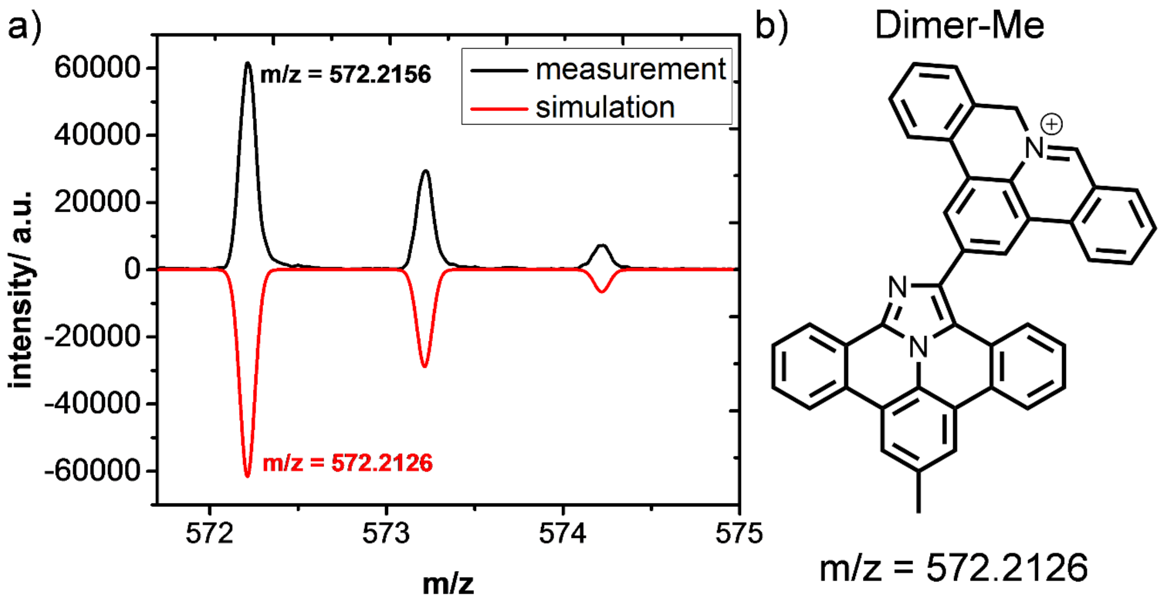

Supplementary Figure 9. **(a)** HR-MALDI-TOF measurement of Dimer-Me (black line) agrees with the expected isotopic distribution pattern (red line). **(b)** Proposed structure of Dimer-Me.

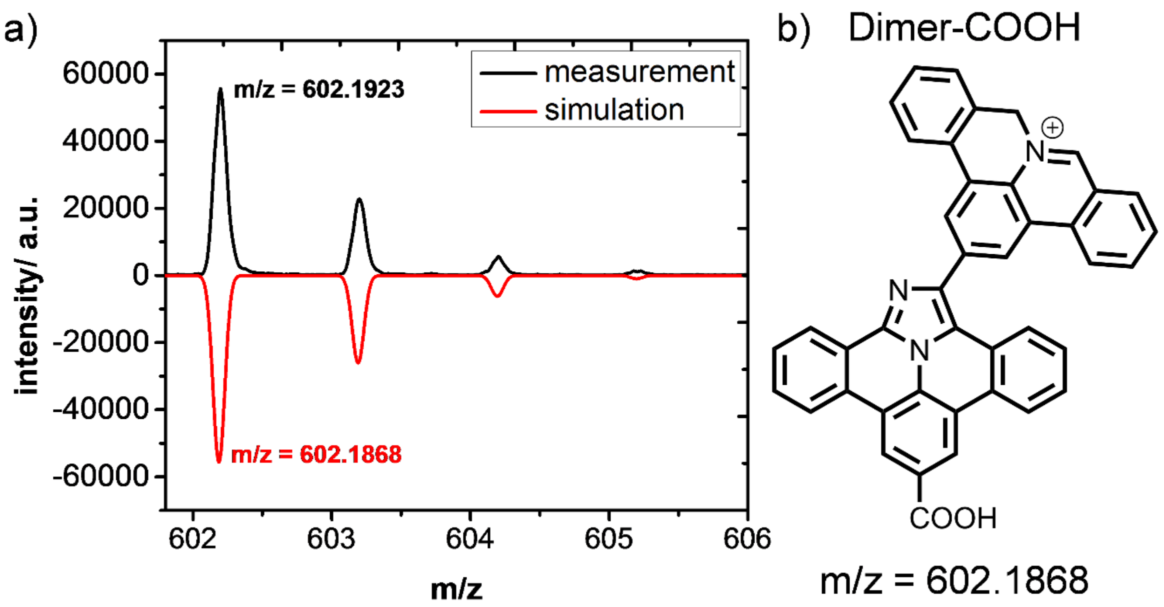

Supplementary Figure 10. **(a)** HR-MALDI-TOF measurement of Dimer-COOH (black line) is in agreement with the expected isotopic distribution pattern (red line). **(b)** Proposed structure of Dimer-COOH.

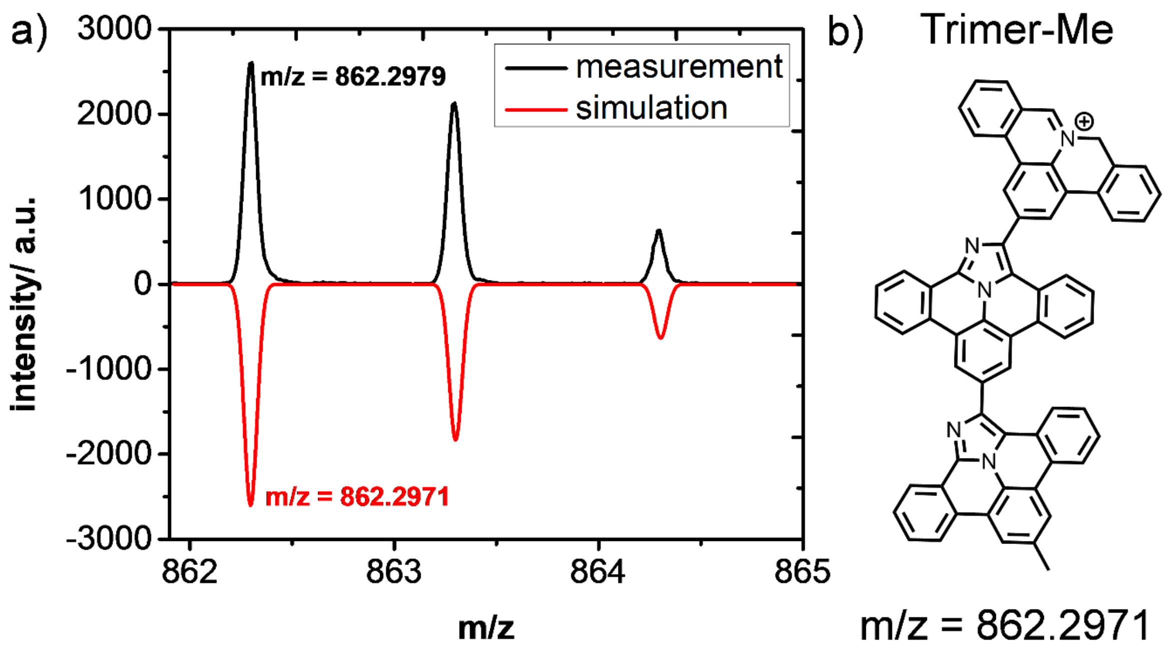

Supplementary Figure 11. **(a)** HR-MALDI-TOF measurement of Trimer-Me (black line) agrees with the expected isotopic distribution pattern (red line). **(b)** Proposed structure of Trimer-Me.

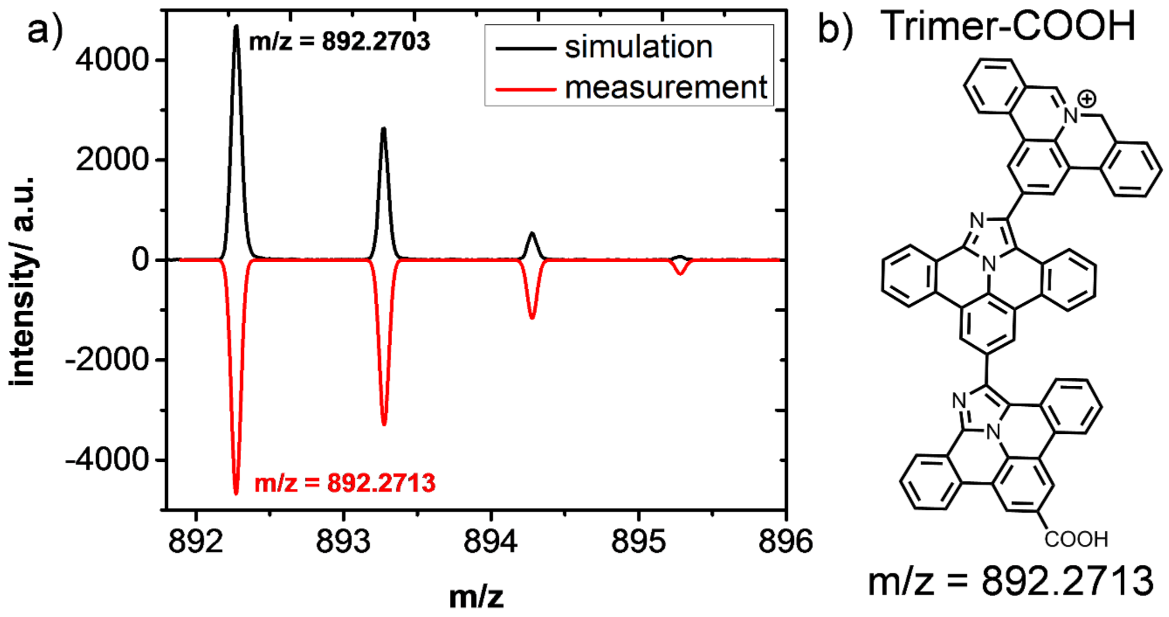

Supplementary Figure 12. **(a)** HR-MALDI-TOF measurement of Trimer-COOH (black line) agrees with the expected isotopic distribution pattern (red line). **(b)** Proposed structure of Trimer-COOH.

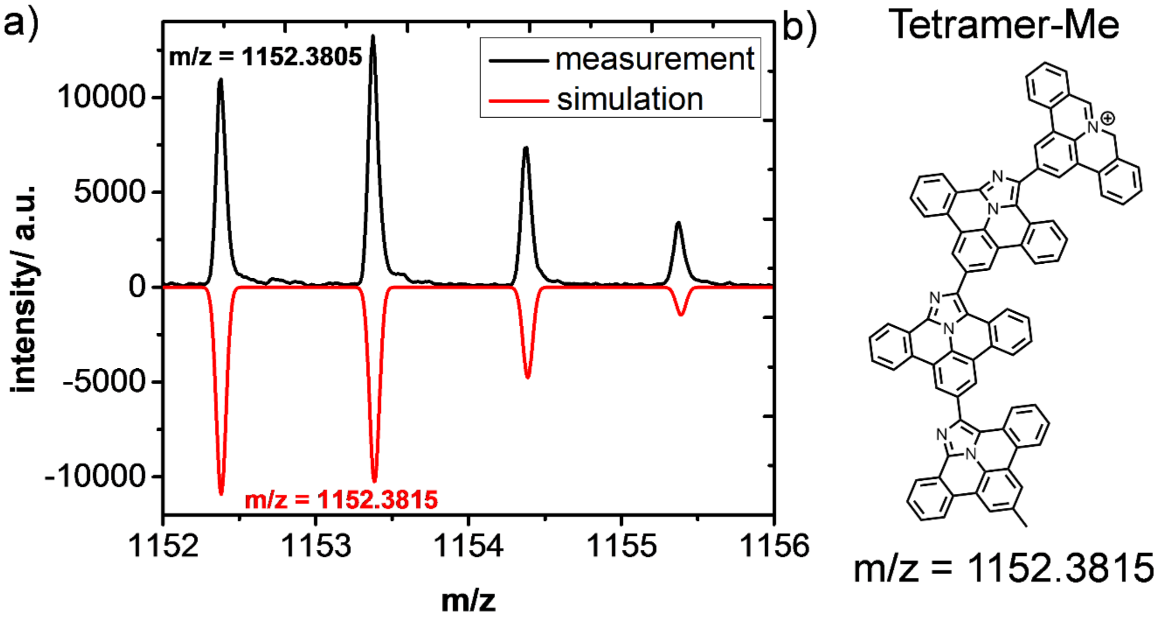

Supplementary Figure 13. **(a)** HR-MALDI-TOF measurement of Tetramer-Me (black line) is in agreement with the expected isotopic distribution pattern (red line). **(b)** Proposed structure of Tetramer-Me.

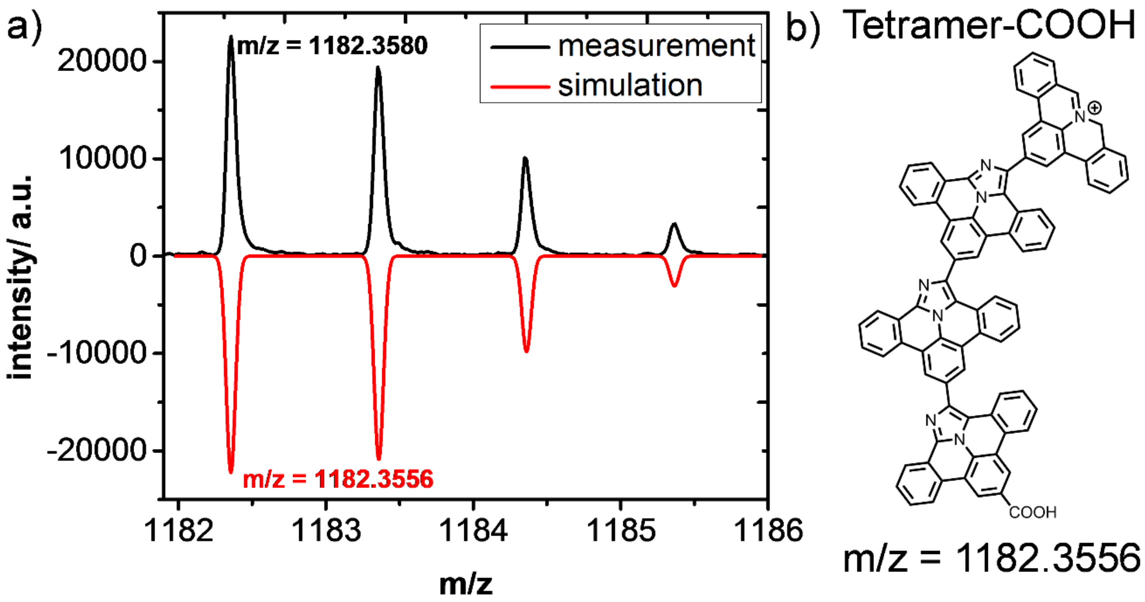

Supplementary Figure 14. **(a)** HR-MALDI-TOF measurement of Tetramer-COOH (black line) is in agreement with the expected isotopic distribution pattern (red line). **(b)** Proposed structure of Tetramer-COOH.

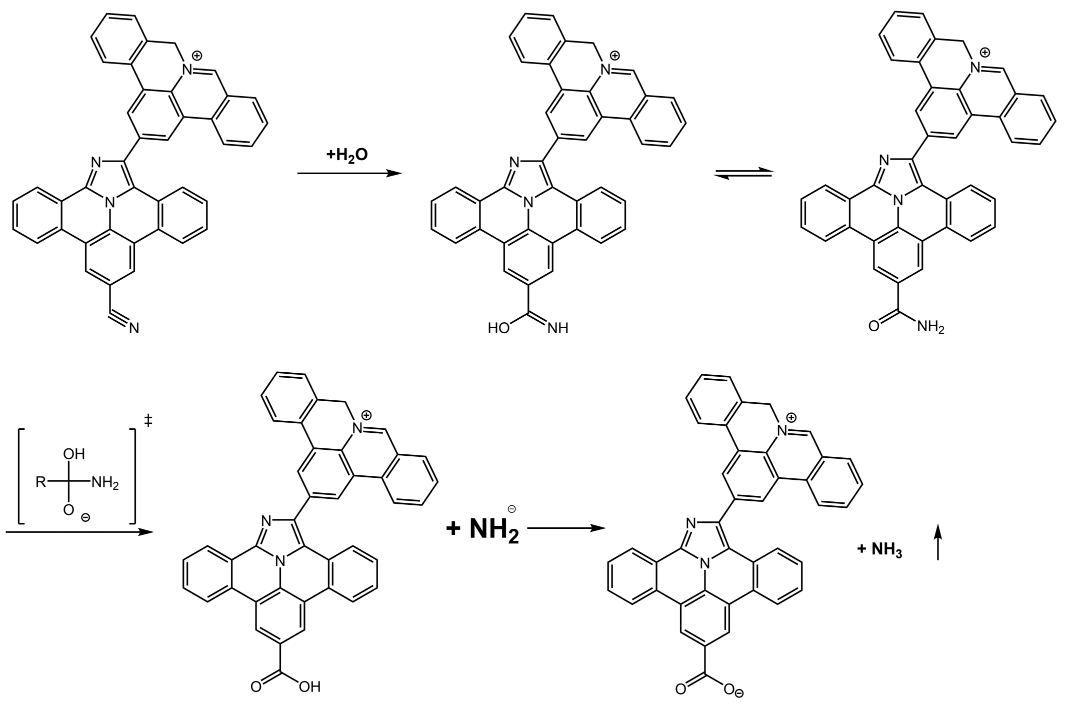

Supplementary Figure 15. Proposed hydrolysis mechanism of the CN-tail of a dimer towards a –COOH group.[47] Indications of –COOH groups were seen in the MALDI spectra after the solid state reaction. No indication of –COOH groups was found for the UHV experiments.

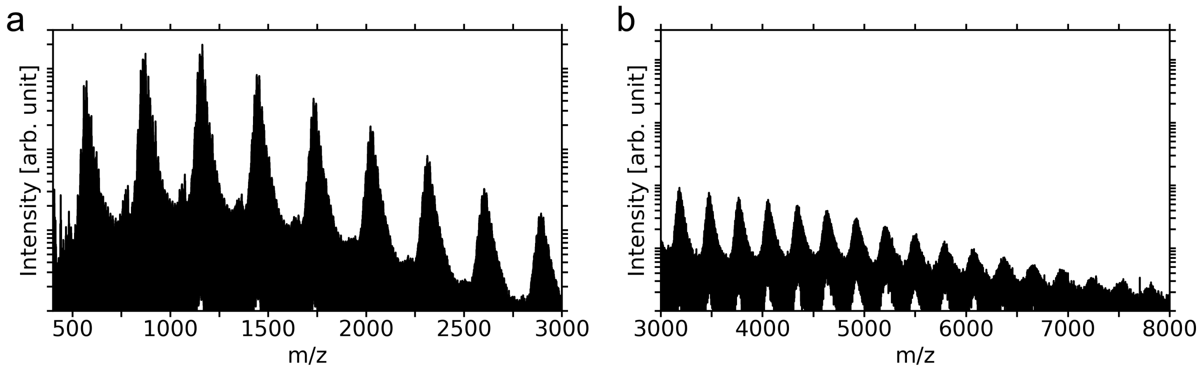

Supplementary Figure 16. MALDI-ToF spectrum after the reaction of a multilayer of **1** at 720 K under 1 mbar Argon carrier gas, shows the formation of oligomer chains with lengths beyond 20 coupled units. **(a)** Spectrum up to m/z=3000 showing a 2893 m/z signal assigned to a 10-mer (the calculated mass for $C_{20}H_{13}N(C_{20}NH_{10}CN)_9$ peaks at 2879.86). **(b)** Spectrum for the high m/z range from m/z=3000 to m/z=8000 showing up to a 7816 m/z signal assigned to a 27-mer (the calculated mass for $C_{20}H_{13}N(C_{20}NH_{10}CN)_{26}$ peaks at 7815.31).

Supplementary Methods

All slab calculations used the PBE exchange-correlation functional.[23] Since we are dealing with physisorbed molecules, we included van der Waals (vdW) corrections via the Grimme's D3 method.[24,25] We investigated the adsorption of dimer (of chemical formula $C_{42}H_{23}N_4$, see Fig. 2h in the main text) on Au(111) surfaces and on h-BN sheets.

The Au(111) surface was modeled with the coordinates derived from the experimental lattice constant of 4.0782 Å. The low adsorbate coverage limit was investigated using 9x9x4 slabs (324 Au atoms, 81 atoms per monolayer) that feature lateral adsorbate separations of almost 26 Å between the centers of masses. The computational unit cell was [(25.9535,0.0,0.0), (12.9768,22.4764,0.0), (0.0,0.0,30.0)] Å. The mean vertical separation between adsorbate and Au top layer was 3.40 Å. The Ag(111) surface was modeled with a 9x9x4 frozen slab with the coordinates derived from the experimental lattice constant of silver (4.0853 angstroms).

We also investigated the physisorption on h-BN sheets. To this end, we generated a 10x10 h-BN sheet ($B_{100}N_{100}$) using the experimental B-N distance of 1.445685 Å. The computational unit cell was [(12.52,21.6853,0.0), (-12.52,21.6853,0.0), (0.0,0.0,21.6832)] Å. The mean vertical separation between adsorbate and h-BN was 3.19 Å. All atoms were relaxed for the adsorbate on the h-BN sheet, whereas only the adsorbate and top Au layer were allowed to relax for adsorption on the Au(111). Geometry relaxations were stopped once all ionic forces fell below 0.025 eV/Å. Surface dipole corrections and over 15 Å of vacuum were used to decouple the images along the non-periodic direction.

For slab calculations, we used the Quickstep (QS) module[26] of the CP2K code[27] [version 3.0, revision number: 16458]. QS solves the electronic problem using a hybrid basis set approach that combines Gaussian and plane wave basis sets. The valence Kohn-Sham orbitals were expanded in a double-zeta basis sets DZVP-MOLOPT-GTH (MOLOPT-DZVP-SR-GTH for Au atoms), which is specifically optimized for its use with the GTH pseudopotentials.[28,29] The valence electronic density was expanded using a plane wave cutoff of 600 Ry. All CP2K calculations were carried out at the Gamma K point. Additionally, we checked our CP2K slab calculations with the projector augmented wave pseudo-potential method (PAW)[30] as implemented in the VASP code[31–34] (version: 5.4.1, 05Feb16). The convergence threshold of the electronic cycle was set to 10^{-5} eV and a Gaussian smearing of 0.1 eV was used. The kinetic energy cutoff was 400 eV and a 5x5x1 K point mesh was used. All gas phase calculations were done at the B3LYP/def2-TZVP35[35] level of theory, supplemented with the D3BJ Grimme's vdW corrections using the ORCA code[36] (version 4.0.0.2). The charge transfers were computed via the Bader analysis code by Henkelman et al. [37–39]

STM images were simulated under the Tersoff-Hamann S-wave tip approximation[40] as implemented in the VASP and CP2K codes. Also, the ASE[41] together with GPAW[42,43] code were used. AFM simulations were performed with the "Probe Particle Model" of Hapala et al.[44,45] using a quadrupole dz^2 tip model, 0.5 N/m bending stiffness, effective charge of -0.25 e, and amplitude of 1.0 Å. These simulation parameters were similar to Ref. [46].

The DFT reaction energies for the dimerization from **1a** to **2a** were calculated by subtracting the dimer **2a** energies from the monomer **1a** energies on frozen metal surfaces and on a relaxed h-BN sheet (see Fig. 7 in the main text): $\Delta E_{dimerizaton} = E_{\mathbf{2a},ads} + E_{empty\ slab} - 2 \cdot E_{\mathbf{1a},ads}$

Supplementary References

1. Baker Cortés, B. D. & Stöhr, M. Role of cyano groups in the self-assembly of organic molecules on metal surfaces. in *Encyclopedia of Interfacial Chemistry* 153–165 (Elsevier, 2018). doi:10.1016/B978-0-12-409547-2.13540-1.

2. Lepper, M. *et al.* Adsorption behavior of a cyano-functionalized porphyrin on Cu(111) and Ag(111): From molecular wires to ordered supramolecular two-dimensional aggregates. *J. Phys. Chem. C* **121**, 26361–26371 (2017).

3. Pacchioni, G. E., Pivetta, M. & Brune, H. Competing interactions in the self-assembly of NC-Ph3-CN molecules on Cu(111). *J. Phys. Chem. C* **119**, 25442–25448 (2015).

4. Krotzky, S. *et al.* Thermodynamics of the segregation of a kinetically trapped two-dimensional amorphous metal–organic network. *J. Phys. Chem. C* **120**, 4403–4409 (2016).

5. Lepper, M. *et al.* Controlling the self-metalation rate of tetraphenylporphyrins on Cu(111) via cyano functionalization. *Angew. Chemie Int. Ed.* **57**, 10074–10079 (2018).

6. Schlickum, U. *et al.* Metal−organic honeycomb nanomeshes with tunable cavity size. *Nano Lett.* **7**, 3813–3817 (2007).

7. Klyatskaya, S. *et al.* Surface-confined self-assembly of di-carbonitrile polyphenyls. *Adv. Funct. Mater.* **21**, 1230–1240 (2011).

8. Pawin, G. *et al.* A surface coordination network based on substrate-derived metal adatoms with local charge excess. *Angew. Chemie Int. Ed.* **47**, 8442–8445 (2008).

9. Fendt, L.-A. *et al.* Modification of supramolecular binding motifs induced by substrate registry: Formation of self-assembled macrocycles and chain-like patterns. *Chem. - A Eur. J.* **15**, 11139–11150 (2009).

10. Faraggi, M. N. *et al.* Bonding and charge transfer in metal–organic coordination networks on Au(111) with strong acceptor molecules. *J. Phys. Chem. C* **116**, 24558–24565 (2012).

11. Sk, R. *et al.* Enhancing intermolecular interaction by cyano substitution in copper phthalocyanine. *J. Phys. Chem. C* **122**, 429–437 (2018).

12. Stöhr, M. *et al.* Self-assembly and two-dimensional spontaneous resolution of cyano-functionalized [7]helicenes on Cu(111). *Angew. Chemie Int. Ed.* **50**, 9982–9986 (2011).

13. Wintjes, N. *et al.* Supramolecular synthons on surfaces: Controlling dimensionality and periodicity of tetraarylporphyrin assemblies by the interplay of cyano and alkoxy substituents. *Chem. - A Eur. J.* **14**, 5794–5802 (2008).

14. Klappenberger, F. *et al.* Uniform π-system alignment in thin films of template-grown dicarbonitrile-oligophenyls. *Adv. Funct. Mater.* **21**, 1631–1642 (2011).

15. Wood, P. A., Borwick, S. J., Watkin, D. J., Motherwell, W. D. S. & Allen, F. H. Dipolar C≡N...C≡N interactions in organic crystal structures: database analysis and calculation of interaction energies. *Acta Crystallogr. Sect. B Struct. Sci.* **64**, 393–396 (2008).

16. Schlickum, U. *et al.* Chiral kagomé lattice from simple ditopic molecular bricks. *J. Am. Chem. Soc.* **130**, 11778–11782 (2008).

17. Schulz, F. *et al.* Elemental identification by combining atomic force microscopy and Kelvin probe force microscopy. *ACS Nano* **12**, 5274–5283 (2018).

18. Glatzel, T. *et al.* Oriented growth of porphyrin-based molecular wires on ionic crystals analysed by nc-AFM. *Beilstein J. Nanotechnol.* **2**, 34–39 (2011).

19. Gutzler, R. *et al.* Surface mediated synthesis of 2D covalent organic frameworks: 1,3,5-tris(4-bromophenyl)benzene on graphite(001), Cu(111), and Ag(110). *Chem. Commun.* **0**, 4456 (2009).

20. Liu, M. *et al.* Thermally induced transformation of nonhexagonal carbon rings in graphene-like nanoribbons. *J. Phys. Chem. C* **122**, 9586–9592 (2018).

21. Shiotari, A. *et al.* Strain-induced skeletal rearrangement of a polycyclic aromatic hydrocarbon on a copper surface. *Nat. Commun.* **8**, 16089 (2017).

22. Li, L., Reich, S. & Robertson, J. Defect energies of graphite: Density-functional calculations. *Phys. Rev. B* **72**, 184109 (2005).

23. Perdew, J. P., Burke, K. & Ernzerhof, M. Generalized gradient approximation made simple. *Phys. Rev. Lett.* **77**, 3865–3868 (1996).

24. Grimme, S., Antony, J., Ehrlich, S. & Krieg, H. A consistent and accurate *ab initio* parametrization of density functional dispersion correction (DFT-D) for the 94 elements H-Pu. *J. Chem. Phys.* **132**, 154104 (2010).

25. Grimme, S., Ehrlich, S. & Goerigk, L. Effect of the damping function in dispersion corrected density functional theory. *J. Comput. Chem.* **32**, 1456–1465 (2011).

26. VandeVondele, J. *et al.* Quickstep: Fast and accurate density functional calculations using a mixed Gaussian and plane waves approach. *Comput. Phys. Commun.* **167**, 103–128 (2005).

27. Hutter, J., Iannuzzi, M., Schiffmann, F. & VandeVondele, J. cp2k: atomistic simulations of condensed matter systems. *Wiley Interdiscip. Rev. Comput. Mol. Sci.* **4**, 15–25 (2014).

28. Goedecker, S., Teter, M. & Hutter, J. Separable dual-space Gaussian pseudopotentials. *Phys. Rev. B* **54**, 1703–1710 (1996).

29. Hartwigsen, C., Goedecker, S. & Hutter, J. Relativistic separable dual-space Gaussian pseudopotentials from H to Rn. *Phys. Rev. B* **58**, 3641–3662 (1998).

30. Blöchl, P. E. Projector augmented-wave method. *Phys. Rev. B* **50**, 17953–17979 (1994).

31. Kresse, G. & Hafner, J. *Ab initio* molecular dynamics for liquid metals. *Phys. Rev. B* **47**, 558–561 (1993).

32. Kresse, G. & Joubert, D. From ultrasoft pseudopotentials to the projector augmented-wave method. *Phys. Rev. B* **59**, 1758–1775 (1999).

33. Kresse, G. & Furthmüller, J. Efficient iterative schemes for *ab initio* total-energy calculations using a plane-wave basis set. *Phys. Rev. B* **54**, 11169–11186 (1996).

34. Kresse, G. & Hafner, J. Norm-conserving and ultrasoft pseudopotentials for first-row and transition elements. *J. Phys. Condens. Matter* **6**, 8245–8257 (1994).

35. Weigend, F. & Ahlrichs, R. Balanced basis sets of split valence, triple zeta valence and quadruple zeta valence quality for H to Rn: Design and assessment of accuracy. *Phys. Chem. Chem. Phys.* **7**, 3297 (2005).

36. Neese, F. The ORCA program system. *Wiley Interdiscip. Rev. Comput. Mol. Sci.* **2**, 73–78 (2012).

37. Tang, W., Sanville, E. & Henkelman, G. A grid-based Bader analysis algorithm without lattice bias. *J. Phys. Condens. Matter* **21**, 084204 (2009).

38. Sanville, E., Kenny, S. D., Smith, R. & Henkelman, G. Improved grid-based algorithm for Bader charge allocation. *J. Comput. Chem.* **28**, 899–908 (2007).

39. Henkelman, G., Arnaldsson, A. & Jónsson, H. A fast and robust algorithm for Bader decomposition of charge density. *Comput. Mater. Sci.* **36**, 354–360 (2006).

40. Tersoff, J. & Hamann, D. R. Theory of the scanning tunneling microscope. *Phys. Rev. B* **31**, 805–813 (1985).

41. Hjorth Larsen, A. *et al.* The atomic simulation environment - a Python library for working with atoms. *J. Phys. Condens. Matter* **29**, 273002 (2017).

42. Mortensen, J. J., Hansen, L. B. & Jacobsen, K. W. Real-space grid implementation of the projector augmented wave method. *Phys. Rev. B* **71**, 035109 (2005).

43. Larsen, A. H., Vanin, M., Mortensen, J. J., Thygesen, K. S. & Jacobsen, K. W. Localized atomic basis set in the projector augmented wave method. *Phys. Rev. B* **80**, 195112 (2009).

44. Hapala, P. *et al.* Mechanism of high-resolution STM/AFM imaging with functionalized tips. *Phys. Rev. B* **90**, 085421 (2014).

45. Hapala, P., Temirov, R., Tautz, F. S. & Jelínek, P. Origin of high-resolution IETS-STM images of organic molecules with functionalized tips. *Phys. Rev. Lett.* **113**, 226101 (2014).

46. Kawai, S. *et al.* Multiple heteroatom substitution to graphene nanoribbon. *Sci. Adv.* **4**, eaar7181 (2018).

47. Izzo, B., Harrell, C. L. & Klein, M. T. Nitrile reaction in high-temperature water: Kinetics and mechanism. *AIChE J.* **43**, 2048–2058 (1997).
